# Socioeconomic, institutional, environmental, and demographic determinants of population health in 90 developing countries: panel data evidence

**DOI:** 10.3389/fpubh.2026.1838213

**Published:** 2026-09-18

**Authors:** Tahsin Tabassum Ahmed, Md. Golam Kibria, Mohammad Ridwan, Jeremy Ko, Chun Kai Leung, K. P. Jaheer Mukthar, Arsene Mouongue Kelly, Md. Akter Hossain, Chunlan Guo, Wai-kit Ming

**Affiliations:** 1 Department of Economics, Noakhali Science and Technology University, Noakhali, Bangladesh; 2 Department of Economics and Banking, International Islamic University Chittagong, Chittagong, Bangladesh; 3 ARUCAD Research Centre, Arkin University of Creative Arts and Design, Nothern Cyprus, Türkiye; 4 Center for Comparative and International Studies, ETH Zurich, Zurich, Switzerland; 5 Erasmus School of Social and Behavioural Sciences, Erasmus Rotterdam University, Rotterdam, Netherlands; 6 Global Society and Sustainability Lab, University of Hong Kong, Hong Kong, Hong Kong SAR, China; 7 Department of Economics, Kristu Jayanti (Deemed to be University), Bengaluru, India; 8 Statistic and Population Studies Department, University of the Western Cape, Cape Town, South Africa; 9 Center for Studies and Research in Economics and Management (CERME), University of Dschang, Dschang, Cameroon; 10 Collaborating Centre for Oxford University and CUHK for Disaster and Medical Humanitarian Response (CCOUC), The Chinese University of Hong Kong, Hong Kong, Hong Kong SAR, China; 11 Department of Infectious Diseases and Public Health, City University of Hong Kong, Hong Kong, Hong Kong SAR, China

**Keywords:** ecological footprint, economic misery, human capital, infant mortality, institutional quality, life expectancy, maternal mortality, population health

## Abstract

Anchored in Grossman’s health production framework, this study examines how socioeconomic, institutional, environmental, and demographic conditions are associated with population health in developing countries. Population health is measured through three complementary indicators—life expectancy, infant mortality, and maternal mortality—to capture broader survival conditions, early-life vulnerability, and maternal health risks. Using panel data for 90 developing countries from 2002 to 2018, the study estimates country fixed-effects models and uses Driscoll–Kraay standard errors to strengthen inference in the presence of heteroskedasticity, autocorrelation, and cross-sectional dependence. Additional robustness checks are conducted using Panel-Corrected Standard Errors, Feasible Generalized Least Squares, and winsorized specifications. The results show that human capital, government effectiveness, and population density are consistently associated with higher life expectancy and lower infant and maternal mortality, and that these coefficient signs remain stable across robustness checks. Economic misery is associated with lower life expectancy and higher mortality in the baseline models, although its significance weakens in some sensitivity tests. Government health expenditure and educational attainment are generally associated with more favorable health outcomes, but their estimates are less stable after winsorization. Ecological footprint yields mixed associations across the three health indicators, while political stability produces unstable and, in some cases, counterintuitive coefficients. Overall, the findings suggest that population health in developing countries is associated more consistently with human capability and institutional effectiveness than with health expenditure alone.

## 1 Introduction

Population health remains a central development concern in many developing countries. Although life expectancy has improved over time in much of the developing world, these gains have often coexisted with persistently high levels of infant and maternal mortality. This pattern suggests that improvements in survival are uneven across population groups and stages of life, and that health outcomes should not be understood through a single indicator alone (1, 2). In this study, population health is examined through three complementary measures—life expectancy, infant mortality, and maternal mortality—so that broader survival conditions, early-life vulnerability, and maternal health risks can be considered together. This broader perspective is especially relevant in developing-country settings, where improvements in average longevity may coexist with persistent weaknesses in maternal care, child survival, nutrition, sanitation, and access to basic health services (3, 4). Accordingly, population health is understood here as national-level health performance reflected jointly in longevity, infant survival, and maternal survival.

Understanding why health outcomes differ across developing countries requires attention not only to healthcare provision, but also to the wider structural conditions within which health is produced. Anchored in Grossman’s (5) health production framework, this study proceeds from the view that population health is shaped by a combination of direct inputs and broader enabling conditions. From this perspective, socioeconomic resources, environmental pressures, institutional quality, and demographic context may all be associated with how effectively countries convert available means into longer survival and lower mortality (6, 7). This framework provides a basis for examining multiple determinants jointly rather than treating them as isolated influences. It also reflects the broader insight that health outcomes are embedded in systems of education, governance, economic conditions, and environmental exposure rather than being determined by medical care alone.

This study asks three related questions. First, how are human capital, economic misery, government health expenditure, ecological footprint, government effectiveness, political stability, educational attainment, and population density associated with life expectancy in developing countries? Second, how are these same factors associated with infant mortality? Third, do the observed associations differ when maternal mortality is used as the health outcome? These questions are examined using panel data for 90 developing countries over the period 2002–2018. The sample is determined by the availability of comparable annual data across the variables included in the analysis, allowing the estimation of a balanced macro-panel for countries classified as emerging and developing economies. The focus on developing countries is substantively important because the determinants of health are often shaped there by greater institutional constraints, wider deficits in access to basic services, higher exposure to economic vulnerability, and more persistent infant and maternal mortality risks than is typically the case in developed countries (8, 9). As a result, these countries provide a particularly relevant setting in which to examine how socioeconomic, institutional, environmental, and demographic conditions are associated with population health outcomes.

Human capital and education are often linked to better health knowledge, preventive behavior, and more effective use of healthcare services (10, 11). Economic misery may weaken household capacity to maintain nutrition, treatment, and basic living conditions (12, 13). Government health expenditure reflects public commitment to health provision, while government effectiveness and political stability capture aspects of the institutional environment within which health systems operate (14, 15). Ecological footprint represents environmental pressure that may shape the conditions under which health is sustained, and population density captures a demographic context that may affect both access to services and congestion-related risks (16, 17). Considered together, these variables reflect a broader view of health production in which population health depends on more than medical care alone.

This study contributes to the literature in three ways. First, it adopts a broader outcome framework by examining life expectancy, infant mortality, and maternal mortality together rather than relying on a single proxy for health performance. Second, it brings together socioeconomic, environmental, institutional, and demographic determinants within one empirical specification, thereby reflecting more closely the multidimensional logic of the Grossman framework. Third, it provides panel evidence for 90 developing countries over 2002–2018, adding comparative evidence for a large cross-country sample of developing economies. The study does not propose a new theory of health production, but offers a more integrated empirical application of an established framework across multiple health indicators. Empirically, the analysis uses fixed-effects models to account for unobserved country-specific heterogeneity, while Driscoll–Kraay standard errors are used to strengthen inference in the presence of heteroskedasticity, autocorrelation, and cross-sectional dependence. Additional robustness checks are conducted using Panel-Corrected Standard Errors, Feasible Generalized Least Squares, and winsorized specifications. Even so, the study does not claim definitive causal identification, and the estimated coefficients are interpreted as conditional associations rather than conclusive causal effects. The findings are therefore presented as cross-country panel associations within the observed sample and period.

The remainder of the paper is structured as follows. Section 2 reviews the literature and develops the hypotheses within the health production framework. Section 3 describes the data, variables, and empirical methodology. Section 4 presents the empirical results and robustness analysis. Section 5 discusses the findings, implications, limitations, and directions for future research.

### 1.1 Literature review

Population health can be understood more coherently through Grossman’s (5) health production framework, which conceptualizes health as a form of capital that contributes to healthy time, productivity, and overall well-being. Although the framework was originally developed at the individual level, it has since been extended to macro-level analysis, where population health is treated as the outcome of how societies translate available resources and institutional capacity into longer survival and lower mortality (6, 7). From this perspective, health outcomes depend not only on medical care, but also on wider social, economic, environmental, institutional, and demographic conditions.

Within this framework, human capital and education can improve health by strengthening knowledge, preventive behavior, and the effective use of healthcare services (5, 10, 11). Government health spending affects health more directly through the provision of medical care, public health services, and health infrastructure (18, 19). Economic misery, by contrast, may weaken health by reducing purchasing power and deepening material deprivation, while ecological pressure may worsen the environmental conditions that sustain health (20). Institutional factors such as government effectiveness and political stability may also shape how successfully available resources are converted into health improvements, since weak institutions and instability can disrupt policy implementation and service delivery (21). Population density adds a further contextual dimension, as concentration may improve access to services in some settings while increasing congestion and pressure on living conditions in others (17).

Within this extended Grossman-type perspective, life expectancy, infant mortality, and maternal mortality reflect related but distinct dimensions of population health. Life expectancy captures the cumulative survival outcome associated with broader social, economic, and institutional conditions. Infant mortality is more sensitive to failures in early-life care, nutrition, sanitation, and basic health services, whereas maternal mortality reflects how effectively health systems and surrounding socioeconomic conditions protect women during pregnancy and childbirth (1, 2). These indicators are therefore better understood as complementary rather than interchangeable, since each captures a different dimension of overall health performance. Taken together, they provide a broader basis for examining how the selected determinants are associated with longevity, child survival, and maternal health.

### 1.2 Human capital and health outcome nexus

Within Grossman’s (5) health capital framework, human capital constitutes a core input in the production and maintenance of health because it increases the efficiency with which individuals generate health over time. More broadly, human capital embodies a stock of knowledge, skills, abilities, and productive capacity that shapes how people manage health-related decisions across the life course. Individuals with higher human capital are generally better able to process information, assess risk, adopt preventive behavior, and use medical care more effectively. It can also improve how time and other resources are allocated, thereby helping to preserve health and slow the depreciation of health capital (22). On this basis, stronger human capital would be expected to contribute to better population health outcomes by improving the conversion of available inputs into health gains. The empirical literature is largely in line with this expectation. In Bangladesh, for example, human capital investment has been found to improve life expectancy, suggesting that the accumulation of human capability supports better long-run health outcomes (10). There is also evidence that the relationship may operate in both directions, as higher longevity can in turn promote human capital formation over time, pointing to a mutually reinforcing connection between health and human capital accumulation across countries (11).

Evidence from other contexts points in a similar direction, though the strength of the relationship appears to depend on wider developmental conditions. Research on oil-abundant African economies shows that human capital improves healthy life expectancy, especially in the long run (22). Other recent studies indicate that digitalization, government effectiveness, public education expenditure, GDP per capita, and urbanization all contribute to human capital accumulation, underscoring the fact that human capability is shaped by broader structural conditions (18, 22). Although these studies are more directly concerned with the determinants of human capital than with health outcomes themselves, they nonetheless support the broader view that human capital is a cumulative productive asset with important implications for health performance. Taken together, the literature generally suggests that human capital is positively associated with health outcomes. Accordingly, the following hypothesis is proposed:

*H1*: Human capital is positively associated with health outcomes.

### 1.3 Economic misery and health outcome nexus

From a Grossman-type health production perspective, economic misery represents a condition that weakens the capacity of individuals and households to produce and maintain health over time. High inflation, unemployment, and broader economic instability reduce real purchasing power, limit access to food, medicine, and healthcare, and increase uncertainty in everyday decision-making. Its effect on health therefore extends beyond income loss alone to the erosion of the material and psychological conditions needed to sustain health capital. Under persistent economic strain, households have fewer resources to devote to nutrition, preventive care, and treatment, while stress and insecurity may further accelerate the depreciation of health (12, 23). In principle, then, greater economic misery should be associated with poorer population health outcomes. Available evidence broadly supports this view. Rising economic misery has been shown to reduce life expectancy in the long run, indicating that inflationary pressure and worsening economic conditions can undermine health performance over time (13). Comparable evidence from MENA economies also points to a negative and statistically significant effect of economic distress on longevity, reinforcing the argument that macroeconomic hardship can weaken health outcomes across countries (24). Findings from Nigeria similarly suggest that poverty shocks worsen mortality conditions and shorten life expectancy, consistent with the idea that economic hardship constrains the ability to sustain health capital (73).

The broader literature indicates that these adverse effects are observable in different settings, even if their magnitude varies across structural contexts. Cross-country evidence shows that higher economic misery is linked to higher infant mortality, while research from India identifies a long-run relationship between economic distress and falling life expectancy (25, 26). More recent studies likewise report that poverty significantly lowers life expectancy in developing and emerging economies and is associated with worse child health outcomes among poorer populations (12, 27). Some studies do note that the relationship may not be perfectly linear in every case, especially under unusual macroeconomic or crisis conditions (23, 28). Even so, the balance of evidence points to a negative relationship between economic misery and health performance. Accordingly, the literature suggests that economic misery is negatively associated with health outcomes.

*H2*: Economic misery is negatively associated with health outcomes.

### 1.4 Government health spending and health outcome nexus

In a Grossman-type framework, government health spending functions as an important public input in the production and maintenance of health. Through the financing of medical services, preventive care, health personnel, and basic health infrastructure, public expenditure can expand access to healthcare and improve the quality of service delivery. In this way, it supports the accumulation of health capital by enabling individuals to prevent illness, obtain treatment when needed, and avoid premature mortality over time (14). As such, higher government health spending would be expected to improve health outcomes. This expectation is broadly supported in the empirical literature. Evidence from Pakistan shows that health expenditure improves life expectancy and reduces infant mortality in both the short and long run (29). Similar findings suggest that domestic health spending contributes positively to life expectancy while lowering infant mortality, reinforcing the beneficial role of health investment in improving population health (14). Maitra (10) likewise reports a long-run relationship between public health spending and longevity, indicating that the effects of such expenditure may persist over time.

Studies from a wider range of countries largely reach the same conclusion, though the size of the effect appears to depend on institutional and structural conditions. Evidence from developing Asian countries links higher health expenditure with lower infant mortality and higher life expectancy (30). More recent research also points in the same direction. In African countries, greater public health expenditure is associated with lower under-five mortality (19), and Bunyaminu et al. (31) find that government health expenditure improves life expectancy, albeit with variation depending on governance quality. Likewise, Khan et al. (32) report a positive long-run effect of government health expenditure on life expectancy in Pakistan, and Anwar et al. (33) show that higher public health spending improves both life expectancy and infant mortality outcomes in OECD countries. To be sure, some evidence suggests that higher spending does not automatically guarantee better outcomes where inefficiencies persist (34). Nevertheless, the broader literature supports a positive relationship between government health spending and health performance.

*H3*: Government health spending is positively associated with health outcomes.

### 1.5 Ecological footprint and health outcome nexus

Ecological footprint can be understood within a Grossman-type health production framework as a form of environmental pressure that may weaken the broader conditions needed to sustain health. A rising ecological footprint generally signals greater pressure on natural resources, ecological imbalance, and environmental degradation, all of which can harm air quality, water quality, food systems, and other living conditions that support health. Its influence on health operates not simply through individual behavior, but through the broader environmental setting within which health capital is accumulated and sustained (35). As environmental pressure intensifies, exposure to pollution, climate-related stress, and ecological disruption may rise, increasing mortality risk and undermining overall well-being. Viewed in this way, a larger ecological footprint would be expected to correspond to worse population health outcomes. The available evidence is broadly consistent with this expectation. Cross-country evidence shows that environmental degradation affects life expectancy, suggesting that ecological deterioration contributes to differences in health outcomes across nations (16). Similar results from Eastern Europe indicate that ecological footprint increases infant mortality, reinforcing the view that environmental pressure worsens population health conditions (36). Findings from Asian economies also show that ecological footprint damages the ecosystem and raises infant mortality, consistent with the idea that environmental stress weakens the health production environment.

At the same time, the literature suggests that the magnitude—and in some cases even the direction—of this relationship may depend on structural conditions and time horizon. Evidence from E7 economies, for instance, suggests that ecological footprint may be associated with higher life expectancy in the short run but lower life expectancy in the long run, pointing to a more complex temporal relationship between environmental pressure and health performance (37). Despite this nuance, more recent studies largely support the harmful long-run effect of ecological degradation on health. In Ethiopia, ecological footprint is associated with lower life expectancy and higher infant mortality (35). Comparable findings from selected Asian economies show that a higher ecological footprint worsens under-five and infant mortality outcomes (38). Evidence from MENA countries also indicates that ecological footprint lowers average life expectancy overall, although the effect differs between oil-exporting and non-oil-exporting economies and may become nonlinear at different thresholds of environmental pressure (39, 40). Earlier work similarly notes that a rising environmental burden increases health risks, especially mortality (41). Overall, the literature suggests that ecological footprint is negatively associated with health outcomes.

*H4*: Ecological footprint is negatively associated with health outcomes.

### 1.6 Government effectiveness and health outcome nexus

Government effectiveness, within a Grossman-type health production framework, can be understood as an institutional condition that influences how well available resources are transformed into health improvements. Effective government improves policy implementation, public service delivery, administrative coordination, and the management of public resources, thereby strengthening the broader system through which health is produced and maintained (15). Its influence is not limited to the direct provision of healthcare and related services; it also operates indirectly through institutions that support sanitation, regulation, infrastructure, and social protection (42). Where government effectiveness is stronger, health inputs are more likely to reach intended populations and generate lasting improvements in well-being. For this reason, higher government effectiveness would generally be expected to correspond to better population health outcomes (42). Empirical evidence is largely consistent with this expectation. Recent findings from Sub-Saharan Africa show that stronger institutional quality, including government effectiveness, improves life expectancy and reduces infant, under-five, and non-communicable disease mortality, suggesting that better governance strengthens the functioning of health systems (15). Related evidence from WAEMU countries likewise indicates that public health spending improves life expectancy more effectively when governance quality is stronger, implying that institutional effectiveness conditions the extent to which health expenditure is translated into outcomes (43).

The literature also indicates that the role of government effectiveness is visible across different African settings, particularly in relation to both aggregate health performance and disparities across countries. Evidence from 40 African countries shows that better governance increases the likelihood that countries belong to groups with relatively better health outcomes, while reducing the probability of membership in poorer health-outcome clusters (42). Related work further finds that governance quality is associated with lower disparities in under-five mortality within African regional groupings, suggesting that effective institutions matter not only for average outcomes but also for their distribution (42). On balance, the literature suggests that government effectiveness is positively associated with health outcomes because it improves the efficiency with which public resources and institutional capacities are translated into health gains.

*H5*: Government effectiveness is positively associated with health outcomes.

### 1.7 Political stability and health outcome nexus

Political stability, within a Grossman-type health production framework, can be seen as an institutional condition that supports continuity in the production and maintenance of health. Stable political environments strengthen governments’ ability to implement health policies, sustain healthcare services, and manage public resources consistently (44). In this way, political stability contributes to health by preserving the continuity of health programs and the broader institutional setting within which health outcomes are produced. Where political conditions are more stable, health systems are generally better able to function effectively and deliver sustained improvements in well-being. On this basis, higher political stability would be expected to be associated with better population health outcomes. The empirical literature broadly supports this expectation (45). Earlier studies show that political instability is associated with poorer health outcomes and wider health inequalities, suggesting that unstable governance weakens population health performance (21). Similar evidence from Indonesia indicates that political structures significantly affect longevity, while findings from Comoros show that recurrent political unrest weakens health policy formulation and administrative efficiency, thereby worsening health performance (44, 46).

Further evidence suggests that the effects of political stability remain visible across different settings, although their magnitude may vary with institutional and structural conditions. Comparative studies from Europe indicate that stronger political and governance structures are associated with better life expectancy outcomes (45, 47). Research from Sub-Saharan Africa similarly shows that political factors significantly influence child survival and longevity, with instability linked to worse health outcomes (48). More recent work reinforces this broader pattern. Evidence from Peru suggests that political and institutional instability can weaken the continuity of child health interventions (49). Likewise, evidence from Somalia indicates that political instability may undermine governance, disrupt health services, and reduce the effectiveness of available resources, even where the direct long-run coefficient is not statistically significant (50). Other studies further suggest that more stable institutional conditions strengthen the extent to which broader economic gains and crisis-response capacity are converted into better life expectancy outcomes (51, 52). Taken together, the literature suggests that political stability is positively associated with health outcomes.

*H6*: Political stability is positively associated with health outcomes.

### 1.8 Education and health outcome nexus

Within a Grossman-type health production framework, education is a key input that improves the efficiency with which individuals produce and maintain health over time. Higher educational attainment enhances the ability to acquire and process health-related information, adopt preventive behavior, and make more effective use of healthcare services. Its influence therefore extends beyond knowledge acquisition alone to better decision-making, healthier lifestyles, and more efficient allocation of household resources. Individuals with more education are thus generally better positioned to sustain health and reduce avoidable risks (53, 54). As such, higher education attainment should be associated with better population health outcomes. The empirical record strongly supports this expectation. Evidence from OECD countries shows that each additional year of education is associated with improved health outcomes, highlighting the close link between educational attainment and well-being (55). Other findings indicate that children of mothers with secondary education experience lower infant mortality than those of mothers without formal schooling, underlining the importance of education for early-life health outcomes (3). Although some studies note that the strength of this association varies across populations and methods, the broader evidence remains supportive of a positive education–health nexus (56).

This relationship is also evident across a range of country settings, even though its strength may differ depending on broader developmental conditions. Cross-country evidence shows that higher educational attainment is closely associated with longer life expectancy, particularly where countries have already reached relatively higher levels of survival (57). Similar findings indicate that female education is linked with broader improvements in life expectancy and overall well-being across countries (58). More recent studies further show that education and health tend to improve together over time, as reflected in evidence from Vietnam and in cross-country analyses covering both developed and developing economies (53, 54). Related work from OECD countries also finds that higher educational attainment is associated with lower infant mortality, higher life expectancy, stronger vaccination outcomes, and fewer potential years of life lost (59). Overall, the literature suggests that education is positively associated with health outcomes.

*H7*: Education is positively associated with health outcomes.

### 1.9 Population density and health outcome nexus

Population density, within a Grossman-type health production framework, can be viewed as a contextual factor that influences the conditions under which health is produced and maintained. The concentration of population influences access to infrastructure, healthcare, sanitation, housing conditions, and environmental quality, all of which affect the efficiency with which individuals and communities sustain health. Its effects may operate through both beneficial and adverse channels (17). On the one hand, higher density can improve access to health services and public infrastructure; on the other, it can increase congestion, pollution, disease transmission, and pressure on basic services where planning is weak (60). The expected relationship between population density and health outcomes is therefore unlikely to be uniformly positive or negative. Existing evidence reflects this mixed theoretical expectation. Earlier studies identified a long-run relationship between population density and life expectancy, suggesting that demographic concentration has measurable effects on health over time (61). Other evidence similarly shows that demographic characteristics, including density and population growth, significantly shape life expectancy across regions (62). By contrast, evidence from Metro Vancouver indicates that higher population density may reduce longevity, while rural–urban comparisons suggest that life expectancy can be higher in less densely populated settings (63, 64).

The literature also shows that the health effects of density depend heavily on sanitation, infrastructure, and the broader urban environment. Cross-country evidence from 151 countries indicates that higher population density is associated with higher life expectancy and lower infant and under-five mortality, particularly in developing countries where denser populations may improve access to healthcare and public services (17). However, other studies find that density can worsen health where sanitation is inadequate, as poor sanitation in densely populated areas is linked to higher infant mortality and worse child health outcomes (65). Related evidence suggests that the health implications of dense urban settings are conditioned by surrounding environmental and social factors, including air quality, mobility, green space, healthcare access, and socioeconomic conditions (66). A broader review likewise concludes that the association between urban density and child health is mixed and highly context-specific (60). Evidence from Latin American cities further shows that health outcomes vary considerably across urban areas and are strongly shaped by modifiable city-level conditions such as education, sanitation, overcrowding, and violence (67). Taken together, the literature suggests that population density has a significant but context-dependent association with health outcomes.

*H8*: Population density is significantly associated with health outcomes.

### 1.10 Literature gap and contribution

Grounded in Grossman’s (5) health production framework, this study begins from the premise that population health is shaped by a combination of socioeconomic, environmental, institutional, and demographic conditions, rather than by medical inputs alone. From this perspective, a first gap in the literature concerns the measurement of health outcomes. Much of the cross-country evidence relies on a single indicator—most commonly life expectancy—to represent population health performance (10, 54, 58). Although life expectancy is an important summary measure of survival conditions, it does not fully capture dimensions of health production that are more directly reflected in early-life survival and maternal care. Infant mortality is more sensitive to factors such as nutrition, sanitation, immunization, and access to basic healthcare, while maternal mortality reflects the capacity of health systems, together with surrounding social conditions, to protect women during pregnancy and childbirth (1–3). To address this gap, the present study adopts a broader health-outcome framework by examining life expectancy, infant mortality, and maternal mortality jointly, thereby providing a more comprehensive assessment of population health in developing countries.

A second gap concerns the specification of health determinants in the empirical literature. The Grossman framework implies that health outcomes result from the combined influence of multiple inputs and enabling conditions. However, previous studies have often examined human capital and education (11, 68), economic hardship (13, 24), ecological pressure (16), or governance and political conditions (21, 44, 48) in separate strands of research or within relatively narrow empirical specifications. Consequently, there remains limited integrated evidence on how a broader set of economic, social, environmental, institutional, and demographic factors is associated with health outcomes within a single cross-country framework. The contribution of the present study is therefore modest but meaningful: it brings together human capital, economic misery, government health expenditure, ecological footprint, government effectiveness, political stability, educational attainment, and population density within a common empirical specification. In doing so, the analysis reflects more closely the multidimensional logic of health production in the Grossman tradition.

A third gap relates to empirical coverage in developing countries. Existing studies have generated valuable insights for particular determinants, health outcomes, or regions, but cross-country evidence remains relatively limited when it comes to jointly examining multiple health indicators alongside a broader set of structural determinants within a single macro-level health production framework for developing economies (6, 7, 14). This study addresses that gap by providing panel evidence for 90 developing countries over the period 2002–2018. Its contribution is not to advance a new theory of health production, but rather to offer a more integrated empirical application of the Grossman framework across three complementary health indicators and a wider set of explanatory variables. In this sense, the study makes a modest but useful contribution by consolidating several strands of the literature within a single panel setting for developing countries and by providing additional evidence on the structural correlates of life expectancy, infant mortality, and maternal mortality.

## 2 Data and methodology

### 2.1 Study setup and sample selection

This study uses panel data for 90 countries classified as emerging and developing economies over the period 2002–2018 to examine the determinants of population health outcomes. The use of this classification provides a clearer and more consistent basis for sample selection and is in line with existing studies [e.g., Ko and Dodoo (8); Ko et al. (69)]. The sample is determined by the availability of cross-national time-series data for the variables included in the analysis, while the full list of sample countries is presented in Table A1.

More specifically, the sample includes countries categorized as emerging and developing economies for which the required variables are available for at least part of the study period. To construct a balanced panel, missing observations within otherwise usable country series were completed through interpolation. This procedure was applied only where sufficient data points were available to maintain continuity in the annual series. Since the variables used in this study are annual macro-level indicators that generally evolve gradually over time, interpolation was used to reduce the loss of observations and improve comparability across countries. By contrast, countries with extensive, systematic, or persistent missing values across key variables were excluded from the sample, as interpolation would not provide a reliable basis for data completion in such cases. This issue is particularly relevant for countries with limited statistical transparency, restricted international reporting, or incomplete recognition across major global databases, where several variables are often entirely unavailable or highly inconsistent. In practice, cases such as North Korea and Palestine were not retained in the final sample because multiple variables contained substantial or continuous missing observations. Thus, the final dataset consists of a balanced panel of 90 emerging and developing economies from 2002 to 2018.

The choice of the period 2002–2018 is also guided by data comparability and consistent annual coverage across the main sources. In particular, the Worldwide Governance Indicators, from which government effectiveness and political stability are drawn, provide more suitable and continuous annual coverage from 2002 onward for the purposes of this study. Starting the sample in 2002 therefore helps ensure that the governance variables are consistently aligned with the other indicators included in the analysis. The endpoint of 2018 is likewise determined by the need to maximize balanced coverage across all variables and countries, since extending the sample beyond 2018 would introduce a greater number of missing observations for some indicators and countries across the combined databases used in the study. As a result, post-2018 coverage becomes less consistent and would weaken the completeness and comparability of the balanced panel. As such, restricting the analysis to 2002–2018 therefore improves the comparability, completeness, and internal consistency of the final panel.

### 2.2 Variables and data sources

Table 1 reports the definitions, units of measurement, and data sources for all variables used in the analysis. The study operationalizes population health outcomes using three complementary indicators: life expectancy, infant mortality, and maternal mortality. These measures are selected because they capture distinct yet related dimensions of health performance at the national level. Life expectancy reflects the overall survival conditions of the population, while infant mortality and maternal mortality more directly indicate the quality of child health, maternal care, healthcare access, and the broader effectiveness of public health systems. The combined use of these three indicators therefore provides a more comprehensive representation of population health than reliance on a single proxy alone. It also helps ensure that the empirical findings are not driven by one particular measure of health outcomes, but are instead evaluated across multiple dimensions of national health performance. This approach is consistent with recent studies that employ several health indicators to capture population health more comprehensively [e.g., Leung et al. (9) and Tang et al. (17)].

**Table 1:** Variable details.

Variables	Details	Unit of measurement	Source
LE	Life Expectancy	Life expectancy at birth, total (years)	WDI
IM	Infant Mortality	Infant mortality rate	WDI
MM (ln)	Maternal Mortality (ln)	Maternal mortality ratio (modeled estimate, per 100,000 live births), log-transformed	WDI
HCI	Human Capital	Index based on years of schooling and returns to education	PWT
EM	Economic Misery	Sum of unemployment rate (% of total labor force) and inflation rate (annual %)	WDI
GHE	Domestic Health Expenditure	Domestic general government health expenditure as a percentage of GDP	WDI
PE	Educational Attainment	Share of the population ages 25 and above that has completed at least primary education (%)	WDI
EF (ln)	Ecological Footprint (ln)	Total ecological footprint measured in global hectares (gha), log-transformed	GFN
Govt. EF	Governance Effectiveness	Governance estimate ranging approximately from −2.5 to 2.5, with higher values indicating better government effectiveness	WGI
PS	Political Stability	Political stability estimate ranging approximately from −2.5 to 2.5, with higher values indicating greater political stability	WGI
PD (ln)	Population Density (ln)	People per square kilometer of land area, log-transformed	WDI

The explanatory variables are selected to reflect the main socioeconomic, environmental, institutional, and demographic dimensions emphasized in the health production framework. Human Capital Index is included to capture the contribution of accumulated education and productive capacity to health improvement. Economic Misery, measured as the sum of inflation and unemployment, is used to reflect macroeconomic hardship and its implications for household welfare and access to health-related resources. Government Health Expenditure is included to represent the role of public-sector commitment to healthcare provision. Ecological Footprint captures environmental pressure and resource use, which may affect health through pollution, ecological stress, and long-term sustainability challenges. Government Effectiveness and Political Stability are used to reflect institutional quality and the broader governance environment within which health systems operate. Primary Educational Attainment serves as an indicator of basic educational achievement, which is closely linked to health awareness, preventive behavior, and access to opportunities. Population Density is included to capture demographic concentration, which may shape health outcomes through service accessibility, congestion effects, and settlement patterns.

With respect to data sources, life expectancy, infant mortality, maternal mortality, government health expenditure, primary educational attainment, population density, inflation, and unemployment are obtained from the World Development Indicators (WDI). Data on the Human Capital Index are drawn from the Penn World Table (PWT). Ecological footprint data are collected from the Global Footprint Network (GFN), while government effectiveness and political stability are taken from the Worldwide Governance Indicators (WGI). Economic misery is constructed following Okun’s concept as the sum of inflation and unemployment.

The World Development Indicators provide the major share of the dataset and serve as the principal source for the core health, education, macroeconomic, and demographic variables used in the study. The Penn World Table contributes the Human Capital Index, the Global Footprint Network provides the ecological footprint measure, and the Worldwide Governance Indicators supply the institutional indicators. Maternal mortality, ecological footprint, and population density are transformed into logarithmic form in the empirical analysis to reduce skewness and improve the comparability of the series across countries. The summary statistics of all variables are reported in Table 2.

**Table 2:** Descriptive statistics.

Variable	Obs.	Mean	Std. Dev.	Min	Max
LE	1,530	67.296	8.135	42.125	80.898
IM	1,530	32.678	22.719	3.500	114.700
MM (ln)	1,530	4.665	1.323	0.693	7.034
HCI	1,530	2.271	0.589	1.088	3.570
EM	1,530	15.051	12.924	−24.960	213.301
GHE	1,530	44.061	18.877	4.156	95.142
PE	1,530	5.304	1.982	1.600	13.677
EF (ln)	1,530	17.071	1.745	12.193	23.749
Govt. EF	1,530	−0.329	0.595	−1.644	1.563
PS	1,530	−0.354	0.792	−2.810	1.387
PD (ln)	1,530	4.137	1.316	0.478	7.556

### 2.3 Empirical framework and model specification

In this framework, health outcomes such as life expectancy and mortality indicators are viewed as outputs produced by a combination of economic, social, demographic, environmental, and institutional inputs. The basic form of the health outcome model may be written as follows in Equation 1:

HO=f(α,D,X) (1)

Where HO denotes health outcomes, α represents the initial health condition, D captures health-related inputs such as healthcare provision, and X denotes other relevant characteristics, including socioeconomic, environmental, demographic, and institutional factors. Although the health production framework originated in micro-level analysis, it has been widely applied in macro-level studies of population health (6, 7). In general, the framework implies that improvements in health-related resources and enabling conditions contribute to better health outcomes. Following this logic, the extended functional form of the model can be expressed as in Equation 2:

$$\mathrm{HO}=f(\begin{array}{l} \text{SOCIOECONF},\text{ENVIRONMENTF},\\ \;\text{INSTITUTIONF},\text{DEMOGRAPHICF} \end{array})$$ (2)

Where SOCIOECONF, ENVIRONMENTF, INSTITUTIONF, and DEMOGRAPHICF denote the socioeconomic, environmental, institutional, and demographic dimensions affecting health outcomes. To examine how these determinants influence population health in 90 developing countries, the study estimates separate empirical equations for life expectancy, infant mortality, and maternal mortality. Since maternal mortality, ecological footprint, and population density are log-transformed, these variables are entered in logarithmic form in the estimated models. The baseline models are specified as follows in Equations 3–5:

For life expectancy:

$$\begin{array}{l} LE_{i,t}=\beta_{1}+\beta_{2}\mathrm{HC}I_{\mathrm{it}}+\beta_{3}EM_{\mathrm{it}}+\beta_{4}\mathrm{GH}E_{\mathrm{it}}+\beta_{5}EF_{\mathrm{it}}\\ +\beta_{6}\text{GovtE}F_{\mathrm{it}}+\beta_{7}PS_{\mathrm{it}}+\beta_{8}PE_{\mathrm{it}}+\beta_{9}PD_{\mathrm{it}}+\mu_{\mathrm{it}}\; \end{array}$$ (3)

or infant mortality rate:

$$\begin{array}{l} \mathrm{IM}R_{\mathrm{it}}=\beta_{1}+\beta_{2}\mathrm{HC}I_{\mathrm{it}}+\beta_{3}\;EM_{\mathrm{it}}+\beta_{4}\mathrm{GH}E_{\mathrm{it}}+\beta_{5}EF_{\mathrm{it}}\\ +\beta_{6}\text{GovtE}F_{\mathrm{it}}+\beta_{7}PS_{\mathrm{it}}+\beta_{8}PE_{\mathrm{it}}+\beta_{9}PD_{\mathrm{it}}+\mu_{\mathrm{it}} \end{array}$$ (4)

For maternal mortality rate:

$$\begin{array}{l} \mathrm{MM}R_{\mathrm{it}}=\beta_{1}+\beta_{2}\mathrm{HC}I_{\mathrm{it}}+\beta_{3}\;EM_{\mathrm{it}}+\beta_{4}\mathrm{GH}E_{\mathrm{it}}+\beta_{5}EF_{\mathrm{it}}\\ +\beta_{6}\text{GovtE}F_{\mathrm{it}}+\beta_{7}PS_{\mathrm{it}}+\beta_{8}PE_{\mathrm{it}}+\beta_{9}PD_{\mathrm{it}}+\mu_{\mathrm{it}} \end{array}$$ (5)

Where i denotes countries, t denotes time, $\mu_{i,t}$ is the stochastic error term, and the β are the coefficients to be estimated. Figure 1 illustrates the conceptual relationship between health outcomes and the explanatory variables included in the analysis.

**Figure 1:**
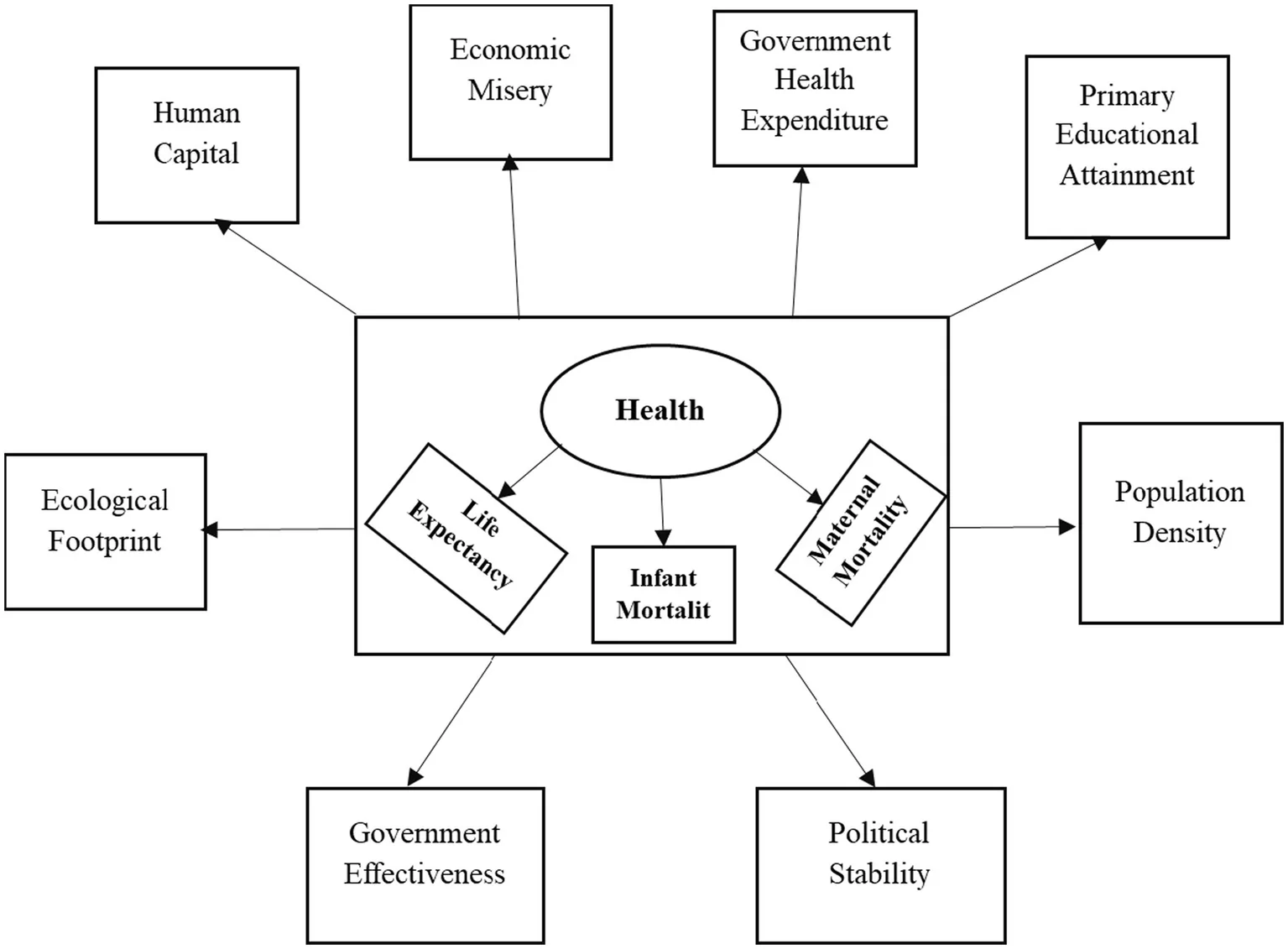
Conceptual framework of determinants of population health.

Regarding the empirical specification (Figure 2), the study first estimates both fixed-effects and random-effects models, and the Hausman test is employed to determine the more appropriate baseline specification. The Hausman test supports the fixed-effects model, as the probability value is below the 5% significance level, implying rejection of the null hypothesis of no systematic difference between the fixed-effects and random-effects estimators. This suggests that the country-specific effects are correlated with the explanatory variables; therefore, the fixed-effects framework is retained for the main analysis. The use of fixed effects is further justified by the likelihood that countries differ in unobserved time-invariant characteristics—such as geography, culture, historical legacies, and structural institutional conditions—that may be correlated with the regressors. Under such circumstances, the fixed-effects estimator provides more reliable coefficient estimates by controlling for country-specific heterogeneity.

**Figure 2:**
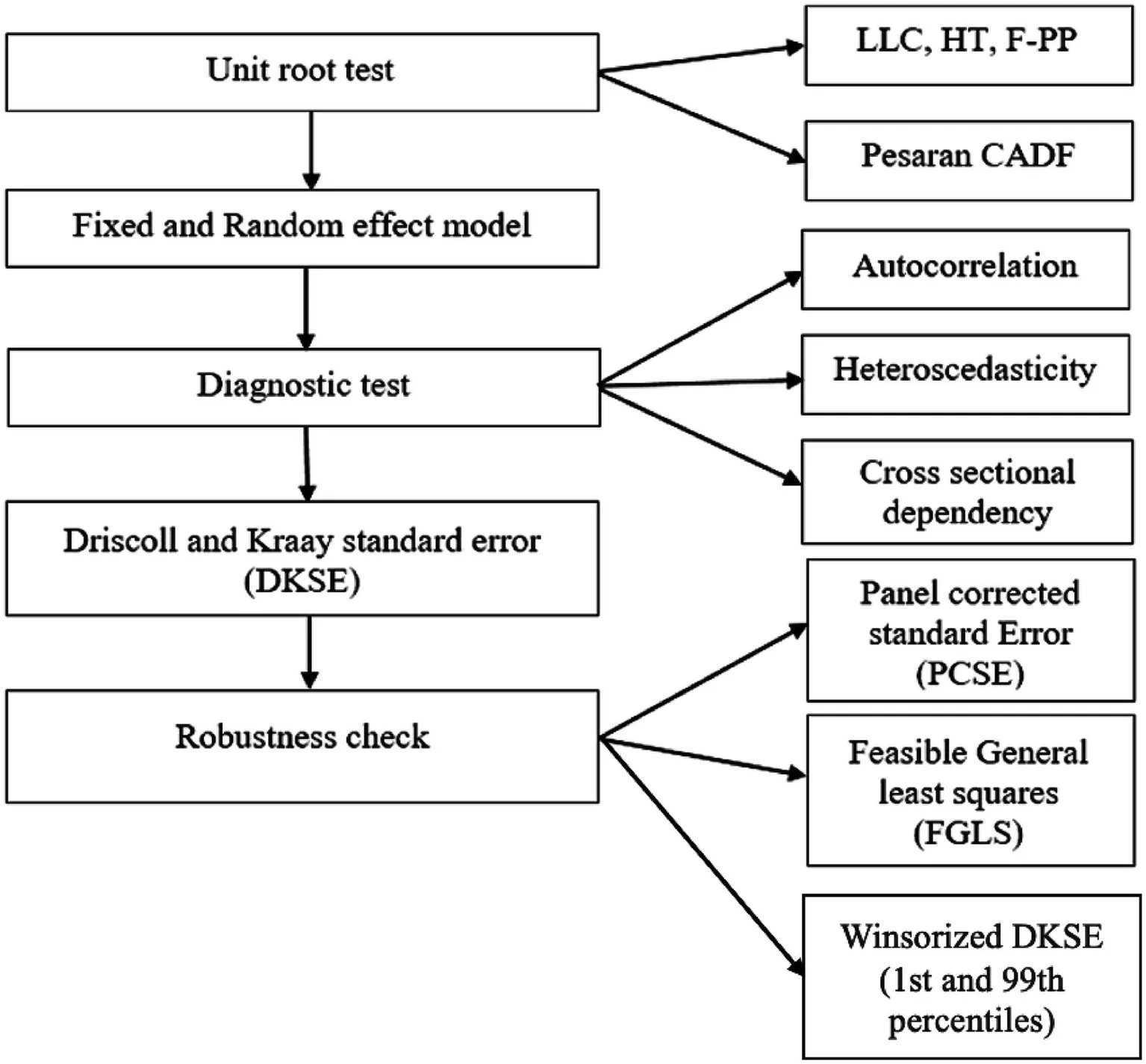
Methodological flow of determinants of population health.

After selecting the baseline specification, diagnostic tests are conducted for heteroskedasticity, serial correlation, and cross-sectional dependence. Since these econometric problems are detected in the data, the study relies primarily on the fixed-effects estimator with Driscoll–Kraay standard errors (DKSE). The DKSE approach is appropriate because it provides standard errors that are robust to heteroskedasticity, autocorrelation, and general forms of cross-sectional dependence, all of which can otherwise bias statistical inference in macro-panel settings. In particular, when countries are interconnected through common shocks, regional spillovers, or global economic conditions, conventional standard errors may understate uncertainty and overstate statistical significance. The DKSE correction is therefore used to obtain more reliable inference for the estimated coefficients. This estimator is also suitable when the cross-sectional dimension exceeds the time dimension, which is consistent with the structure of the present dataset.

To assess the robustness of the results, the study further supplements the DKSE estimates with Panel-Corrected Standard Errors (PCSE) and Feasible Generalized Least Squares (FGLS). PCSE is employed because it is well suited to panel datasets characterized by panel-specific heteroskedasticity and contemporaneous correlation across cross-sectional units, thereby providing an alternative correction for the error structure. FGLS is included as an additional robustness estimator because it explicitly models the variance–covariance structure of the disturbances and may produce more efficient estimates when heteroskedasticity and serial correlation are present. In addition, a sensitivity analysis is conducted by winsorizing the continuous variables at the 1st and 99th percentiles and re-estimating the baseline fixed-effects model with Driscoll–Kraay standard errors. This procedure is used to reduce the influence of extreme observations without excluding them from the sample and to examine whether the estimated associations are sensitive to moderate treatment of outliers. The use of PCSE, FGLS, and the winsorized DKSE specification therefore serves to examine whether the direction, magnitude, and statistical significance of the estimated associations remain stable across alternative estimation strategies and data treatments.

Nevertheless, the estimated coefficients should be interpreted as conditional associations rather than definitive causal effects, since the empirical design does not eliminate all possible sources of endogeneity. In addition, the findings should be interpreted specifically with reference to the sampled panel of developing countries classified as emerging and developing economies included in this study over the period 2002–2018, rather than as universally generalizable relationships applicable to all countries or settings. To summarize the estimation framework in a general form, the baseline panel model underlying the three health-outcome equations may be written as follows in Equation 6:

$$y_{\mathrm{it}}=x\prime_{\mathrm{it}}\beta +\varepsilon_{\mathrm{it}},i=1,\cdots \cdots ,N,t=1,\cdots ..,T\;$$ (6)

Here, $x_{\mathrm{it}}$ denotes the vector of explanatory variables, including human capital, economic misery, government health expenditure, ecological footprint, government effectiveness, political stability, primary educational attainment, and population density. The dependent variable, $y_{\mathrm{it}}$ , denotes the health outcome indicator, namely life expectancy, infant mortality, or maternal mortality. Figure 2 presents the methodological framework of the study. With regard to stationarity, the study reports both first-generation and second-generation panel unit root tests. The LLC, HT, and Fisher-PP tests are presented mainly for comparability with earlier panel-data studies. However, because the diagnostic tests confirm significant cross-sectional dependence across countries, the second-generation Pesaran CADF test is more appropriate for the present dataset. Accordingly, the CADF results reported in Table 3 are given greater weight in determining the order of integration, while the first-generation unit root results are treated as supplementary evidence.

**Table 3:** Second-generation unit root test.

Variables	Pesaran CADF I(0)	Pesaran CADF I(1)
LE	−2.136***	
IM	−2.096***	
MM (ln)	−2.121***	−2.724***
HCI	−3.078***	
EM	−2.555***	
GHE	−2.087***	
PE	−2.042***	
EF (ln)	−2.117***	
Govt. EF	−1.638	−2.876***
PS	−1.829	−2.208***
PD (ln)	−2.320***	−1.634

*, **, and *** indicate significance at the 10, 5, and 1% levels, respectively.

## 3 Results

### 3.1 Panel unit root test results

Before estimating the empirical models, it is necessary to determine the stationarity properties of the panel data, since non-stationary variables may produce spurious regression results and unreliable statistical inference. To examine the order of integration, this study employs three first-generation panel unit root tests: the Levin–Lin–Chu (LLC) test, the Harris–Tzavalis (HT) test, and the Fisher-type Phillips–Perron (Fisher-PP) test. The results are reported in Table 4. Overall, the first-generation unit root tests indicate that the variables are stationary either at level or after first differencing. The LLC results show that all variables are stationary at level except the human capital index, which becomes stationary after first differencing. The HT test provides somewhat stricter evidence, indicating that life expectancy, infant mortality, maternal mortality, human capital, and population density are non-stationary at level but become stationary after first differencing, whereas the remaining variables are stationary in levels. The Fisher-PP test likewise indicates that most variables are stationary at level, although government health expenditure appears to be stationary only after first differencing. Taken together, these findings suggest that the variables are a mixture of I(0) and I(1) processes, with no evidence that any variable is integrated of order two.

**Table 4:** First-generation unit root test.

Variables	LLC I(0)	LLC I(1)	HT I(0)	HT I(1)	Fisher-PP I(0)	Fisher-PP I(1)
LE	−19.6440***		0.9648	−0.1486***	645.5456***	
IM	−42.3506***		0.9280	0.2809***	3232.4144***	
MM (ln)	−4.4249***		0.9448	−0.0077***	163.3181	
HCI	29.1149	−4.4657***	1.0268	0.7229***	364.3938***	
EM	−21.6325***		0.1795***		788.5336***	
GHE	−4.6948***		0.7495***		206.8083*	1441.6774***
PE	−8.7735***		0.8087*	−0.3050***	300.5091***	1931.8055***
EF (ln)	−6.5183***		0.7382***		269.7055***	
Govt. EF	−8.6077***		0.7747***		397.3582***	
PS	−5.8271***		0.8089*		247.4332***	
PD (ln)	−17.2025***	−6.8900***	0.9863	0.5822***	1365.4588***	

*, **, and *** indicate significance at the 10, 5, and 1% levels, respectively.

Because first-generation tests do not explicitly account for cross-sectional dependence, a second-generation unit root test is also conducted using Pesaran’s cross-sectionally augmented Dickey–Fuller (CADF) approach. The CADF results reported in Table 3 are broadly consistent with the earlier findings. Specifically, life expectancy, infant mortality, maternal mortality, human capital, economic misery, government health expenditure, ecological footprint, government effectiveness, and population density are stationary at level. By contrast, political stability and primary educational attainment are non-stationary at level but become stationary after first differencing. Overall, the CADF results confirm that all variables are either I(0) or I(1). This indicates that the dataset is suitable for panel estimation and that the subsequent empirical analysis may proceed without concern over higher-order integration. In substantive terms, the consistency between the first- and second-generation tests strengthens confidence that the estimated associations reported later are not driven by common trending behavior in the data.

### 3.2 Slope heterogeneity test

Given the cross-country structure of the sample, it is also important to determine whether slope coefficients are homogeneous across panel units. To do so, this study applies the slope heterogeneity test proposed by Hashem Pesaran and Yamagata (74). The results are reported in Table 5. Both the Delta and adjusted Delta statistics are positive and statistically significant at the 1% level. Accordingly, the null hypothesis of slope homogeneity is rejected. This finding indicates that the slope coefficients differ across countries, implying that the associations between the explanatory variables and population health outcomes are not uniform for all panel units. In substantive terms, this result is consistent with the expectation that developing countries differ in institutional quality, health-system capacity, demographic structure, and socioeconomic conditions. Therefore, the reported coefficients should be interpreted as average associations across heterogeneous national contexts.

**Table 5:** Slope heterogeneity test.

Test	Delta	*p*-value
Delta	11.103***	0.000
Delta_adj	18.869***	0.000

*** indicate significance at the 10% level.

### 3.3 Model selection and multicollinearity diagnostics

Before discussing the main estimates, baseline regressions were compared using fixed-effects and random-effects specifications. The corresponding results are reported in Table A3. The Hausman specification test supports the fixed-effects model in all three equations, with test statistics of 251.75 for life expectancy, 472.62 for infant mortality, and 181.51 for maternal mortality, each significant at the 1% level. This indicates that the fixed-effects estimator is more appropriate than the random-effects alternative, as the unobserved country-specific effects appear to be correlated with the regressors.

In addition, multicollinearity does not appear to be a serious concern. As reported in Table A2, the variance inflation factor (VIF) values range from 1.06 to 2.44, with a mean VIF of 1.61. These values are below conventional thresholds, suggesting that the explanatory variables do not exhibit problematic collinearity. Based on these diagnostics, the main estimations are reported using the fixed-effects model with Driscoll–Kraay standard errors (DKSE), which is appropriate in the presence of heteroskedasticity, autocorrelation, and cross-sectional dependence. To assess robustness, the results are also compared with estimates obtained from panel-corrected standard errors (PCSE) and feasible generalized least squares (FGLS).

### 3.4 Regression results

Tables 6–8 report the estimated associations of the explanatory variables with life expectancy, infant mortality, and maternal mortality, respectively. Overall, the coefficients are broadly stable across DKSE, PCSE, and FGLS, which strengthens confidence in the empirical findings. As an additional sensitivity check, the baseline fixed-effects models with Driscoll–Kraay standard errors were re-estimated using variables winsorized at the 1st and 99th percentiles, and the results are reported in Table 9. The discussion below therefore considers both the baseline estimates and the extent to which the findings remain sensitive to the treatment of extreme observations.

**Table 6:** Estimation results for life expectancy in developing countries.

Variables	DKSE model	PCSE model	FGLS model
HCI	6.236***	6.236***	6.236***
EM	−0.080***	−0.080***	−0.080***
GHE	0.114***	0.114***	0.114***
PE	0.031	0.031	0.031
EF	0.153*	0.153***	0.153*
Govt. EF	2.436***	2.436***	2.436***
PS	−1.189***	−1.189***	−1.189***
PD	1.287***	1.287***	1.287***
Cons.	41.577***	41.577***	41.577***
Observations	1,530	1,530	1,530
Number of groups	90	90	90
Time periods	17	17	17
R-squared	0.5722	0.5722	—
Root MSE	5.3347	—	—
F/Wald chi2	26432.74	3985.09	2046.74
Prob > F/chi2	0.0000	0.0000	0.0000
Log likelihood	—	—	−4728.042

*, **, and *** indicate significance at the 10, 5, and 1% levels, respectively.

**Table 7:** Estimation results for infant mortality in developing countries.

Variables	DKSE model	PCSE model	FGLS model
HCI	−21.255***	−21.255***	−21.255***
EM	0.118***	0.118***	0.118***
GHE	−0.324***	−0.324***	−0.324***
PE	−1.007***	−1.007***	−1.007***
EF	−0.096	−0.096	−0.096
Govt. EF	−5.090***	−5.090***	−5.090***
PS	0.803*	0.803**	0.803
PD	−2.870***	−2.870***	−2.870***
Cons.	110.900***	110.900***	110.900***
Observations	1,530	1,530	1,530
Number of groups	90	90	90
Time periods	17	17	17
R-squared	0.7031	0.7031	—
Root MSE	12.4121	—	—
F/Wald chi2	4681.90	2832.78	3622.95
Prob > F/chi2	0.0000	0.0000	0.0000
Log likelihood	—	—	−6020.034

*, **, and *** indicate significance at the 10, 5, and 1% levels, respectively.

**Table 8:** Estimation results for maternal mortality in developing countries.

Variables	DKSE model	PCSE model	FGLS model
HCI	−1.262***	−1.262***	−1.262***
EM	0.005**	0.005***	0.005***
GHE	−0.022***	−0.022***	−0.022***
PE	−0.033***	−0.033***	−0.033***
EF	−0.066***	−0.066***	−0.066***
Govt. EF	−0.121*	−0.121***	−0.121***
PS	−0.070	−0.070*	−0.070**
PD	−0.198***	−0.198***	−0.198***
Cons.	10.471***	10.471***	10.471***
Observations	1,530	1,530	1,530
Number of groups	90	90	90
Time periods	17	17	17
*R*-squared	0.7343	0.7343	—
Root MSE	0.6839	—	—
F/Wald chi2	120597.49	71485.53	4228.26
Prob > F/chi2	0.0000	0.0000	0.0000
Log likelihood	—	—	−1585.189

*, **, and *** indicate significance at the 10, 5, and 1% levels, respectively.

**Table 9:** Sensitivity analysis: DKSE estimates using winsorized variables.

Variables	Life expectancy	Infant mortality	Maternal mortality
Human capital	4.869***	−10.704***	−0.634***
Economic misery	−0.017*	0.005	0.002*
Government health expenditure	−0.020**	0.067*	−0.000
Educational attainment	0.157**	−0.301	−0.022***
Ecological footprint (ln)	0.640***	−8.163***	−0.322***
Government effectiveness	0.615***	−2.716***	−0.137***
Political stability	0.245	−0.937	0.032*
Population density (ln)	11.545***	−32.734***	−0.447***
Constant	−1.819	328.985***	13.529***
Observations	1,530	1,530	1,530
Number of groups	90	90	90
*R*-squared	0.5966	0.5945	0.4816

*, **, and *** indicate significance at the 10, 5, and 1% levels, respectively.

#### 3.4.1 Human capital index

Human capital shows one of the most consistent associations across all three health indicators. In the life expectancy model (Table 6), the coefficient on HCI is 6.236 and is statistically significant at the 1% level under DKSE, PCSE, and FGLS. This indicates that a one-unit increase in the human capital index is associated with a 6.236-year increase in life expectancy, holding other variables constant. In the infant mortality model (Table 7), the HCI coefficient is −21.255 and remains significant at the 1% level throughout, indicating that a one-unit increase in human capital is associated with a decline of 21.255 infant deaths per 1,000 live births. A similar pattern is observed in the maternal mortality model (Table 8), where the coefficient is −1.262 and highly significant across all estimators. Since maternal mortality is expressed in logarithmic form, this coefficient indicates that a one-unit increase in human capital is associated with a reduction of 1.262 log points in maternal mortality.

The sensitivity analysis reported in Table 9 confirms that the direction of the HCI coefficient remains unchanged in all three models after winsorization. Although the magnitudes become smaller, the coefficients remain statistically significant at the 1% level in the life expectancy, infant mortality, and maternal mortality models (Table 9), indicating that the association between human capital and population health outcomes is robust to the treatment of extreme observations.

#### 3.4.2 Economic misery

Economic misery also exhibits a clear and stable association across the three baseline models. In the life expectancy model (Table 6), the coefficient on EM is −0.080 and significant at the 1% level across all estimators, indicating that a one-unit increase in the misery index is associated with a 0.080-year decline in life expectancy. In the infant mortality model (Table 7), the coefficient is 0.118 and remains significant at the 1% level, implying that a one-unit increase in the misery index is associated with an increase of 0.118 infant deaths per 1,000 live births. Likewise, in the maternal mortality model (Table 8), the coefficient is 0.005 and is statistically significant, although at a slightly lower level under DKSE. This indicates that a one-unit increase in the misery index is associated with a 0.005 log-point increase in maternal mortality. Overall, higher economic misery is consistently associated with poorer health outcomes in the baseline specifications.

However, the winsorized results in Table 9 suggest that this association is somewhat sensitive to extreme observations. While the coefficient on EM remains negative and weakly significant in the life expectancy model and positive and weakly significant in the maternal mortality model, it becomes statistically insignificant in the infant mortality model (Table 9). This indicates that the estimated association between economic misery and health outcomes weakens after adjusting for outliers.

#### 3.4.3 Government health expenditure

Government health expenditure is also consistently associated with the three health outcomes in the baseline results. In the life expectancy model (Table 6), the coefficient on GHE is 0.114 and statistically significant at the 1% level across all specifications, indicating that a one-percentage-point increase in government health expenditure is associated with a 0.114-year increase in life expectancy. In the infant mortality model (Table 7), the coefficient is −0.324 and remains highly significant, indicating that a one-percentage-point increase in government health expenditure is associated with 0.324 fewer infant deaths per 1,000 live births. In the maternal mortality model (Table 8), the coefficient is −0.022 and again significant at the 1% level in all estimators. Because maternal mortality is log-transformed, this suggests that a one-percentage-point increase in government health expenditure is associated with a 0.022 log-point decline in maternal mortality.

By contrast, the sensitivity analysis in Table 9 indicates that the association for GHE is not robust to winsorization. In the life expectancy model, the coefficient becomes negative and significant; in the infant mortality model, it turns positive and only weakly significant; and in the maternal mortality model, it becomes statistically insignificant (Table 9). These changes suggest that the estimated association between government health expenditure and health outcomes is sensitive to the treatment of extreme observations and should therefore be interpreted with caution.

#### 3.4.4 Primary educational attainment

The association between primary educational attainment and the health outcomes is somewhat mixed across the baseline models. In the life expectancy equation (Table 6), the coefficient on PE is positive (0.031) but statistically insignificant across DKSE, PCSE, and FGLS, indicating that its association with life expectancy is not statistically robust in this specification. However, the variable shows stronger associations in the mortality models. In Table 7, the coefficient is −1.007 and statistically significant at the 1% level in all estimators, indicating that a one-unit increase in primary educational attainment is associated with a decline of 1.007 infant deaths per 1,000 live births. Similarly, Table 8 reports a coefficient of −0.033, also significant at the 1% level throughout, indicating that higher primary educational attainment is associated with lower maternal mortality. These findings suggest that primary educational attainment is more clearly associated with reductions in infant and maternal mortality than with gains in life expectancy.

The winsorized results in Table 9 indicate some weakening of these associations. PE becomes positive and statistically significant in the life expectancy model, loses significance in the infant mortality model, and remains negative and statistically significant in the maternal mortality model (Table 9). Accordingly, the association between primary educational attainment and maternal mortality appears more robust than its associations with life expectancy and infant mortality.

#### 3.4.5 Ecological footprint

The associations involving ecological footprint are less uniform across the three baseline models. In the life expectancy model (Table 6), the coefficient on EF (ln) is 0.153 and is statistically significant at the 10% level under DKSE and FGLS, and at the 1% level under PCSE. Since EF (ln) is log-transformed and life expectancy is measured in levels, a 1% increase in ecological footprint is associated with an approximately 0.00153-year increase in life expectancy. In the infant mortality model (Table 7), the coefficient on EF (ln) is −0.096 but remains statistically insignificant across all estimators, indicating that no robust association is observed between ecological footprint and infant mortality. In the maternal mortality model (Table 8), however, the coefficient is −0.066 and highly significant in all specifications. Because both variables are in logarithms, this coefficient may be interpreted as an elasticity, indicating that a 1% increase in ecological footprint is associated with a 0.066% decline in maternal mortality. Overall, ecological footprint displays mixed associations that vary depending on the health indicator considered.

The sensitivity analysis in Table 9 shows that EF (ln) remains statistically significant across all three winsorized models. Its positive association with life expectancy and negative association with maternal mortality are preserved, while in the infant mortality model the coefficient becomes negative and statistically significant (Table 9). This suggests that the estimated ecological footprint association is not fully stable in the infant mortality model, although it remains directionally favorable for life expectancy and maternal mortality.

#### 3.4.6 Government effectiveness

Government effectiveness is positively associated with better health outcomes across the three baseline models. In the life expectancy model (Table 6), the coefficient on Govt. EF is 2.436 and significant at the 1% level across all estimators, indicating that a one-unit increase in government effectiveness is associated with a 2.436-year increase in life expectancy. In the infant mortality model (Table 7), the coefficient is −5.090 and remains highly significant, indicating that a one-unit increase in government effectiveness is associated with a decline of 5.090 infant deaths per 1,000 live births. In the maternal mortality model (Table 8), the coefficient is −0.121; although its significance is weaker under DKSE than under PCSE and FGLS, the direction remains unchanged across estimators. Overall, these results indicate that stronger government effectiveness is consistently associated with more favorable population health outcomes.

The winsorized results in Table 9 reinforce this general pattern. Govt. EF retains the same signs and remains statistically significant in all three models, although the magnitude of the coefficient is smaller in the life expectancy and infant mortality equations (Table 9). This indicates that the association between government effectiveness and health outcomes is comparatively robust to the treatment of extreme observations.

#### 3.4.7 Political stability

Political stability shows less consistent associations and therefore requires more cautious interpretation. In the life expectancy model (Table 6), the coefficient is −1.189 and statistically significant at the 1% level across all three estimators. This indicates that a one-unit increase in political stability is associated with a 1.189-year decline in life expectancy. In the infant mortality model (Table 7), the coefficient is 0.803; it is significant at the 10% level in DKSE, at the 5% level in PCSE, and becomes statistically insignificant in FGLS. Thus, the estimated positive association between political stability and infant mortality is not fully robust across estimators. In the maternal mortality model (Table 8), the coefficient is −0.070 and its significance varies across specifications. Accordingly, the association between political stability and health outcomes appears to be sensitive to model choice and should be interpreted with caution.

This sensitivity is reinforced by the winsorized estimates in Table 9. In the life expectancy model, the coefficient changes sign and becomes statistically insignificant. In the infant mortality model, the coefficient also changes sign and remains insignificant. In the maternal mortality model, the coefficient becomes positive and weakly significant (Table 9). These shifts indicate that the estimated association for political stability is highly sensitive to the treatment of extreme observations.

#### 3.4.8 Population density

Population density also exhibits a relatively stable association with the health indicators in the baseline models. In the life expectancy model (Table 6), the coefficient on PD (ln) is 1.287 and statistically significant at the 1% level across all estimators. This implies that a 1% increase in population density is associated with an approximately 0.01287-year increase in life expectancy. In the infant mortality model (Table 7), the coefficient is −2.870 and remains significant at the 1% level, indicating that a 1% increase in population density is associated with about a 0.0287 decline in infant mortality. In the maternal mortality model (Table 8), the coefficient is −0.198 and highly significant throughout. Because both variables are in logarithms, this coefficient represents an elasticity, indicating that a 1% increase in population density is associated with a 0.198% decline in maternal mortality. Overall, higher population density is associated with more favorable health outcomes in the estimated models.

The winsorized results in Table 9 preserve this overall pattern. PD (ln) remains positive and highly significant in the life expectancy model and negative and highly significant in both mortality models (Table 9). Although the magnitudes increase substantially after winsorization, the direction and significance of the association remain unchanged, indicating that population density is among the more stable correlates in the analysis.

Overall, the baseline results reported in Tables 6–8 show that the estimated coefficients are broadly stable across DKSE, PCSE, and FGLS, although some variation remains in the significance levels of ecological footprint, political stability, and government effectiveness. The additional sensitivity analysis in Table 9 further indicates that some associations—particularly those for human capital, government effectiveness, and population density—remain relatively robust after winsorization, whereas the estimated associations for economic misery, government health expenditure, primary educational attainment, and political stability are more sensitive to the treatment of extreme observations. The models are based on 1,530 observations from 90 countries over 17 years, and the reported R-squared values for the DKSE and PCSE estimates are 0.5722 for life expectancy, 0.7031 for infant mortality, and 0.7343 for maternal mortality.

### 3.5 Overall patterns and robustness

Overall, the estimates reported in Table 6—reveal several consistent patterns. Human capital, government health expenditure, and government effectiveness are positively associated with improved health outcomes across the three models, while economic misery is associated with poorer outcomes. Primary educational attainment is significantly associated with lower infant and maternal mortality, although its association with life expectancy is not statistically significant. By contrast, the estimated associations for ecological footprint and political stability are less consistent across specifications. The additional sensitivity analysis in Table 9 shows that some of these relationships, especially those involving human capital, government effectiveness, and population density, remain comparatively stable after winsorization, whereas the estimated associations for government health expenditure and political stability are more sensitive to extreme observations.

The results are broadly stable across DKSE, PCSE, and FGLS estimators, which supports the robustness of the main findings. The models are based on 1,530 observations from 90 countries over 17 years, and the reported R2 values indicate moderate explanatory power for life expectancy and relatively stronger explanatory power for infant and maternal mortality.

## 4 Discussion and conclusion

### 4.1 Reasons for the results

Within Grossman’s (5) health production framework, this study examined how socioeconomic, environmental, institutional, and demographic conditions are associated with population health outcomes in developing countries. More specifically, it assessed whether human capital, economic misery, government health expenditure, ecological footprint, government effectiveness, political stability, educational attainment, and population density are associated with variation in life expectancy, infant mortality, and maternal mortality across 90 developing economies over the period 2002–2018. In this framework, health is produced through a combination of direct inputs and broader enabling conditions rather than through medical care alone (5, 7). The results are broadly consistent with that perspective, but they also show that the strength and stability of the associations differ across determinants and across the three health indicators. This suggests that some structural factors are more consistently associated with health outcomes than others.

The findings for human capital provide strong support for H1. Human capital is positively associated with life expectancy and negatively associated with both infant and maternal mortality, and these relationships remain statistically significant after winsorization. One reason for this pattern is that stronger human capital is associated with greater efficiency in the way households and individuals convert available resources into health gains. People with higher knowledge, skills, and productive capacity are generally better able to process health information, recognize risk, adopt preventive behavior, and make more effective use of treatment and healthcare services over time (5, 22). At the macro level, human capital may also reflect a broader developmental capacity associated with healthier working lives, higher productivity, and greater adaptability to health shocks. This helps explain why the coefficient is favorable not only for life expectancy, which reflects cumulative survival conditions, but also for infant and maternal mortality, where timely knowledge and effective interaction with health services are especially important. This result is comparable to Maitra’s (10) evidence for Bangladesh, where human capital investment is positively associated with life expectancy, and to Vu’s (11) cross-country findings, which point to a reinforcing association between health and human capital accumulation. The present estimates therefore suggest that, within developing-country settings, human capital is among the more stable factors associated with health performance across multiple outcome measures.

The results for economic misery generally support H2, although the magnitude of the relationship becomes less stable in the sensitivity analysis. In the baseline estimates, greater economic misery is associated with lower life expectancy and with higher infant and maternal mortality. A plausible explanation is that inflation and unemployment are associated with a weaker ability of households to maintain minimum health-supporting conditions. When prices rise or jobs become insecure, families may cut back on food quality, delay treatment, reduce preventive care, or face greater difficulty paying for transport and medicines. These pressures are particularly consequential for infants and pregnant women because their health depends heavily on continuity of nutrition, early intervention, and regular contact with basic services (12, 23). Economic distress may also be associated with greater psychosocial stress and insecurity, which can weaken the broader environment in which health is maintained (20). The result is broadly comparable to Shahbaz et al. (13), who report that economic misery is negatively associated with life expectancy in Pakistan, and to Alam et al. (25), who find a harmful long-run association between economic distress and health conditions in India. At the same time, the weaker winsorized coefficients suggest that the association is sensitive to extreme observations. For that reason, H2 is supported, but the results indicate that the association between economic misery and health outcomes is less stable once outliers are moderated.

Government health expenditure behaves in a more qualified way and therefore provides only partial support for H3. In the baseline estimates, higher public health expenditure is associated with longer life expectancy and with lower infant and maternal mortality. This is broadly in line with the health production framework, since public spending can expand the availability of medical care, preventive services, skilled personnel, maternal care, and health infrastructure (14, 29). In developing-country settings, public expenditure may be particularly important where private access to healthcare is limited and where the state plays a central role in financing basic health provision. However, the winsorized results show that the coefficients become unstable in sign and significance, suggesting that the estimated relationship is sensitive to extreme observations. This pattern indicates that higher expenditure is not uniformly associated with better health outcomes across the sample. It may reflect cross-country differences in how effectively health resources are allocated and translated into service delivery. This helps explain why the baseline result resembles earlier work showing that public health spending is positively associated with longevity and lower mortality (10, 33), while the weaker sensitivity results are closer to studies emphasizing that expenditure alone is not always associated with better outcomes under institutional inefficiency (34, 43). The evidence therefore suggests that public spending is associated with health outcomes, but that this association is not fully stable across specifications.

The estimates for ecological footprint do not support H4 in a simple or uniform way. The expectation was that greater ecological pressure would be associated with worse health outcomes, yet the observed pattern is mixed. Ecological footprint is positively associated with life expectancy, statistically insignificant for infant mortality in the baseline model, and negatively associated with maternal mortality. In the winsorized specifications, it remains positively associated with life expectancy and becomes negatively associated with both mortality indicators. This mixed pattern suggests that ecological footprint is not uniformly associated with health outcomes across the three indicators. One possible interpretation is that, in developing countries, ecological footprint may capture not only environmental pressure but also aspects of economic activity and resource use that are associated with better infrastructure and living conditions (37, 70). At the same time, the less favorable associations with mortality outcomes are consistent with studies linking environmental stress to poorer health conditions, particularly for more vulnerable populations (16, 36). In this respect, the findings differ from studies that report a more uniformly harmful association between ecological footprint and health outcomes, such as Ayitehgiza et al. (35), and are somewhat closer to Biyase et al. (37), who show that this relationship can vary across contexts and time horizons. H4 is therefore not supported consistently, and the results indicate that the association between ecological footprint and health outcomes is complex rather than uniform.

Government effectiveness provides one of the clearest cases of support for H5. In both the baseline and winsorized models, greater government effectiveness is associated with longer life expectancy and with lower infant and maternal mortality. This result is plausible because government effectiveness shapes whether health-related resources are actually converted into usable services. Where implementation capacity is stronger, public health programs are more likely to function regularly, budgets are more likely to reach facilities, and service delivery is more likely to remain continuous across time and territory (15, 71). Government effectiveness may also be associated with better health through non-medical channels, such as sanitation provision, administrative coordination, regulatory quality, and infrastructure maintenance, which improve the broader conditions under which health is produced (42). This helps explain why the coefficients are favorable across all three health indicators rather than only for one of them. The result is comparable to Sun et al. (15), who show that stronger institutions are associated with higher life expectancy and lower mortality in Sub-Saharan Africa, and to Boundioa and Thiombiano (43), who show that governance quality strengthens the association between health expenditure and longevity. The present results therefore imply that institutional capacity is not merely a background condition; it is one of the potential channels through which other health-supporting inputs become associated with better outcomes.

Political stability, by contrast, does not behave in line with H6 and remains the least stable result in the analysis. In the baseline models, political stability is negatively associated with life expectancy, positively associated with infant mortality, and only weakly associated with maternal mortality. In the winsorized models, the signs shift and the coefficients become even less consistent. These results do not support the conventional expectation that stable political environments should be associated with better health by protecting policy continuity and reducing institutional disruption. One possible reason is that the political stability indicator may not fully capture broader dimensions of governance that are more directly associated with health outcomes, such as policy effectiveness or service delivery capacity. It is also possible that the relationship between political stability and health varies across countries depending on institutional and historical context. The inconsistency of the coefficients further suggests that the estimated association is not robust across specifications. In this sense, the results differ from studies that report a more clearly favorable association between political stability and life expectancy or child survival (45, 48). H6 is therefore not supported. The more cautious conclusion is that political stability, at least as measured here, is not a consistently reliable factor associated with health outcomes across this heterogeneous developing-country sample.

The findings for educational attainment offer partial support for H7 and appear more convincing for mortality outcomes than for life expectancy. In the baseline models, primary educational attainment is not significantly associated with life expectancy, but it is negatively associated with infant and maternal mortality. After winsorization, the effect becomes positive and significant for life expectancy, insignificant for infant mortality, and remains negative and significant for maternal mortality. One possible reason for this pattern is that education is associated with mechanisms that are especially relevant to maternal and early-life health. Educational attainment is often linked to greater literacy, better awareness of hygiene and symptoms, improved ability to follow treatment advice, higher use of antenatal services, and stronger understanding of child nutrition and immunization schedules (3, 54). These mechanisms may help explain why education is more consistently associated with infant and maternal mortality than with life expectancy, which reflects broader and longer-run survival conditions across the population. Education may also be associated with greater autonomy in household decision-making and a higher likelihood of timely care-seeking during pregnancy and childbirth, a relationship long emphasized in the population-health literature (72). The present results are therefore closer to studies that find a particularly strong education association with child and maternal survival than to work focusing only on longevity across the full life course (58, 59). Accordingly, H7 is supported more clearly in relation to maternal mortality, less consistently for infant mortality, and only weakly for life expectancy.

Population density provides strong support for H8 and is among the more robust findings in the study. In the baseline and winsorized estimates, higher population density is associated with longer life expectancy and with lower infant and maternal mortality. Since the hypothesis proposed only that population density would be significantly associated with health outcomes, these findings clearly support H8. One possible explanation is that denser settlement is associated with greater spatial efficiency in service provision. Where populations are more concentrated, the cost of delivering clinics, hospitals, roads, water systems, schools, and health outreach may be lower per person, while physical access to formal care may also be better. These conditions may be particularly relevant for maternal and infant health, where timely access to emergency care, skilled attendance, and routine services is closely associated with survival (17). Denser environments may also be associated with faster information diffusion and more regular interaction with public institutions. This result is consistent with the cross-country findings of Tang et al. (17), who show that higher population density is associated with better health outcomes in many developing settings, but it differs from studies that emphasize the congestion and sanitation risks of dense settlement under weak infrastructure (60, 65). Overall, the results suggest that, in this sample, the advantages associated with service access and concentration are more evident than the disadvantages associated with crowding.

Taken together, the findings show that the hypotheses receive uneven but still informative support. H1, H2, H5, and H8 are broadly supported. H3 and H7 receive partial support. H4 and H6 are not supported consistently by the empirical evidence. More substantively, the most stable results are concentrated in variables that are associated with productive capability, as in the case of human capital, or with the institutional translation of available resources into outcomes, as in the case of government effectiveness. Economic misery is associated with poorer health performance by weakening the material conditions needed to preserve health. Education appears to matter most clearly where health outcomes depend strongly on informed household behavior and timely use of care. Public spending, environmental pressure, and political stability, by contrast, appear to be associated with health outcomes through more conditional and context-sensitive pathways. The overall picture that emerges is therefore not that all determinants matter in the same way, but that health outcomes in developing countries are shaped by a layered production process in which knowledge, institutions, material resources, and surrounding conditions are associated with health in different degrees and through different mechanisms.

### 4.2 Implications

The findings imply that, for policymakers in developing countries, the most credible entry points for improving health outcomes are human capital and government effectiveness. These two variables are the most consistent in their association with higher life expectancy and lower infant and maternal mortality, even after the sensitivity analysis. This means that the policy lesson from the study is not simply that more health inputs are needed, but that population health is more strongly associated with the capacity of people and institutions to make effective use of those inputs. In practical terms, the results support giving sustained priority to policies that strengthen human capability and improve the functioning of public institutions, since these appear more reliably associated with better health outcomes than several of the other determinants examined.

The results also have a more specific implication for health-sector planning. Government health expenditure is associated with better health outcomes in the baseline models, but this relationship becomes unstable in the winsorized analysis. This suggests that policymakers should be cautious about interpreting higher health spending, by itself, as evidence of likely health improvement. The implication is that resource allocation matters, but the effectiveness with which spending is translated into actual services appears to matter at least as much. In that sense, the stronger and more stable role of government effectiveness gives the findings a clear administrative implication: improving service delivery, implementation capacity, and the functioning of public systems may be a more dependable route to better health outcomes than focusing on expenditure expansion alone.

Another implication concerns the design of social policy across health indicators. Educational attainment is more clearly associated with lower infant and maternal mortality than with life expectancy, while economic misery is associated with poorer health outcomes, although less robustly once extreme observations are moderated. This suggests that some determinants are more relevant for specific dimensions of health than for others. For governments and development agencies, the implication is that strategies aimed at reducing infant and maternal mortality should give particular attention to education and to the household conditions that shape access to care and preventive behavior. More broadly, the results suggest that health policy in developing countries may be more effective when it is differentiated according to the type of outcome being targeted rather than assuming that the same factors matter equally for all health measures.

Finally, the findings imply that some commonly discussed structural variables should be treated more cautiously in policy interpretation. Ecological footprint does not show a uniform association across the three health outcomes, and political stability is the least stable result in the analysis. These results suggest that neither variable provides a straightforward policy signal in this study. By contrast, population density shows a robust association with all three outcomes, indicating that the spatial distribution of population may have practical relevance for how health services are accessed and organized. Taken together, the broader implication of the study is that health improvement in developing countries is likely to depend less on any single policy lever and more on whether countries strengthen the particular structural conditions that are most consistently associated with better health outcomes in the evidence presented here.

## 5 Limitations and future research directions

Despite the contributions of this study, several limitations should be acknowledged when interpreting the findings. First, an important limitation concerns endogeneity. Although the analysis employs fixed effects, Driscoll–Kraay standard errors, and additional robustness checks, these methods mainly improve statistical inference in the presence of heteroskedasticity, autocorrelation, cross-sectional dependence, and time-invariant unobserved heterogeneity. They do not fully eliminate endogeneity arising from reverse causality, simultaneity, or omitted time-varying factors. This issue is particularly relevant because some explanatory variables, such as human capital, education, government effectiveness, and health expenditure, may themselves be shaped by prior health conditions. For this reason, the estimated coefficients should be interpreted as conditional associations rather than definitive causal effects. Future research could address this limitation more directly by using stronger identification strategies, such as dynamic panel estimators, instrumental-variable approaches, or other causal designs where suitable data and identification conditions are available.

Relatedly, the scope of the empirical model is necessarily selective. Although the study includes a broad set of socioeconomic, environmental, institutional, and demographic factors, it does not capture all possible determinants of population health. Other relevant influences, such as income inequality, sanitation, access to safe water, immunization coverage, nutritional conditions, disease burden, health workforce availability, and social protection arrangements, are not explicitly incorporated into the specification. As a result, the reported associations should be understood within the bounds of the variables examined here rather than as a complete account of the health production process in developing countries. Future research could build on the present framework by incorporating a wider range of health-system and social determinants in order to assess whether the observed patterns remain stable once these omitted dimensions are taken into account.

In addition to variable coverage, the way the main concepts are measured also places limits on interpretation. Several of the variables used in the analysis are broad proxy indicators, and the results may therefore depend partly on how both the explanatory and dependent variables are operationalized. For example, human capital is measured using the Penn World Table index, educational attainment is represented by completion of at least primary education among adults aged 25 and above, and government health expenditure is measured as domestic general government health expenditure as a percentage of GDP. Likewise, government effectiveness and political stability are captured through broad governance indicators, while ecological footprint reflects general environmental pressure rather than more direct health-relevant exposures such as air pollution, unsafe water, or localized ecological degradation. On the outcome side, life expectancy, infant mortality, and maternal mortality each capture an important dimension of health, but each remains only one particular representation of population health performance. Future research could therefore test the robustness of the findings using alternative measures, including more disaggregated education, expenditure, governance, environmental, and health-system indicators.

A further limitation concerns the functional form of the analysis. The empirical framework adopted here is primarily associational and linear in nature. While this approach is useful for identifying average conditional relationships across a large cross-country panel, the underlying relationships may in fact be more complex than a simple linear specification can capture. In practice, some determinants may operate through nonlinear, threshold, moderated, or interaction-based pathways. For example, the association between health expenditure and health outcomes may depend on institutional quality, while the implications of population density or ecological pressure may vary according to sanitation, infrastructure, or broader development conditions. Examining such mechanisms in a fully developed way is beyond the scope of the present study. The current analysis therefore does not model these more complex pathways directly. Future research could extend the analysis by testing interaction effects, nonlinearities, threshold relationships, or mediation pathways in order to better capture the layered structure of health production. A further limitation follows from the time period covered by the data. Because of data availability and the need to maintain a balanced panel, the analysis is restricted to 2002–2018. While this period is appropriate for the present design, it does not capture the major disruptions associated with the COVID-19 pandemic and its aftermath. The post-2020 period brought substantial changes in health-system pressures, fiscal priorities, labor-market conditions, and patterns of vulnerability across developing countries, all of which may have altered the relationships examined in this study. Future research should therefore revisit these associations using more recent data, especially for the post-COVID period, in order to determine whether the main findings remain stable under more recent health and development conditions.

Finally, beyond issues of identification, variable coverage, measurement, functional form, and time period, the macro-level nature of the analysis also limits what can be said about mechanisms. The study identifies broad cross-country associations, but it cannot directly observe the household, community, service-delivery, or institutional pathways through which these relationships operate. As a result, some of the mechanisms discussed in interpreting the findings remain inferential rather than directly tested within the empirical model. This is especially relevant for variables such as education, government effectiveness, and population density, where the link to health outcomes may run through several intermediate processes. Future research could help open this black box by combining macro-panel evidence with micro-level, household, subnational, or sector-specific data so that the channels connecting structural determinants to health outcomes can be examined more directly.

## Data Availability

The original contributions presented in the study are included in the article/supplementary material, further inquiries can be directed to the corresponding authors.
