# Supplementary material for: Socioeconomic, institutional, environmental, and demographic determinants of population health in 90 developing countries: panel data evidence
Source: Front Public Health. 2026 Sep 18;14:1838213. doi: 10.3389/fpubh.2026.1838213 (PMC13630590; doi:10.3389/fpubh.2026.1838213)
Supplement: Supplementary file 1 [file Table_1.docx]

**Appendices**

| Algeria | Angola | Argentina | Armenia | Bahrain | Bangladesh | Barbados |
| --- | --- | --- | --- | --- | --- | --- |
| Belize | Benin | Bolivia | Botswana | Brazil | Brunei Darussalam | Bulgaria |
| Burkina Faso | Burundi | Cambodia | Cameroon | Chile | China | Colombia |
| Congo, Rep | Costa Rica | Cote d'lvoire | Dominican Republic | Ecuador | Egypt, Arab Rep. | El Salvador |
| Eswatini | Ethiopia | Fiji | Gambia, The | Ghana | Guatemala | Guyana |
| Honduras | Hungary | India | Indonesia | Iran, Islamic Rep. | Jamaica | Kazakhstan |
| Kenya | Kuwait | Kyrgyz Republic | Lao PDR | Lesotho | Madagascar | Malawi |
| Malaysia | Mali | Mauritania | Mauritius | Mexico | Moldova | Mongolia |
| Morocco | Mozambique | Myanmar | Namibia | Nepal | Nicaragua | Niger |
| Nigeria | Pakistan | Panama | Paraguay | Peru | Philippines | Poland |
| Qatar | Romania | Russian Federation | Rwanda | Sao Tome and Principle | Senegal | South Africa |
| Sri Lanka | Tajikistan | Tanzania | Thailand | Togo | Tunisia | Turkey |
| Uganda | Ukraine | Uruguay | Venezuela, RB | Viet Nam | Zambia |  |

**Table A1.** Developing country lists

**Table A2.** Multicollinearity test

| Variables | VIF |  |
| --- | --- | --- |
| Govt. EF | 2.27 | HCI |
| GHE | 1.88 | EM |
| HCI | 1.69 | GHE |
| EF | 1.05 | PE |
| PS | 1.59 | EF |
| PE | 1.10 | Govt. EF |
| EM | 1.05 | PS |
| PD | 1.18 | PD |
| Mean | 1.48 |  |

**Table A3.** Fixed and random effect estimation

| Variables | Life Expectancy | | Infant Mortality | | Maternal Mortality | |
| --- | --- | --- | --- | --- | --- | --- |
|  | FE | RE | FE | RE | FE | RE |
| HCI | 10.547*** | 10.353 | -30.247 | -29.960 | -173.110 | -192.659 |
| EM | -0.027*** | -0.028 | 0.091 | 0.091 | 0.689 | 0.634 |
| GHE | -0.007** | 0.001 | 0.022 | -0.019 | 0.094 | -0.106 |
| EF | 1.030*** | 2.700 | -2.710 | -4.010 | -9.390 | 5.880 |
| Govt. EF | 0.917*** | 0.940 | -3.945 | -3.991 | -26.414 | -31.281 |
| PS | 0.004 | -0.051 | -0.398 | -0.251 | -1.141 | -1.362 |
| PE | 0.221*** | 0.215 | -0.417 | -0.497 | 5.110 | 4.657 |
| PD | 0.010*** | 0.010 | -0.036 | -0.026 | -0.222 | -0.164 |
| Constant | 41.462 | 41.855 | 105.556 | 105.207 | 588.540 | 635.050 |
| Number of Country | 90 | 90 | 90 | 90 | 90 | 90 |
| Diagnostic testing | | | | | | |
| Hausman Test | 14.81** |  | 44.75* |  | 21.71*** |  |
| Heteroskedasticity (Wald Test) | 396097.22*** |  | 595210.81*** |  | 591700.20*** |  |
| Autocorrelation (Wooldridge test ) | 14.915*** |  | 121.741*** |  | 282.516*** |  |
| CSD test (Pesaran’s test) | 12.992*** |  | 19.126*** |  | 0.682* |  |

Note: *, **, and *** representing the level of significance for 10%, 5%, and 1*, respectively
